# Influence of Mothers’ Habits on Reading Skills and Emotional Intelligence of University Students: Relationships in the Social and Educational Context

**DOI:** 10.3390/bs10120187

**Published:** 2020-12-07

**Authors:** Elena Jiménez-Pérez, Almudena Barrientos-Báez, David Caldevilla-Domínguez, José Gómez-Galán

**Affiliations:** 1 Departament of Didactics of Languages, Art, and Sports, University of Malaga, Campus de Teatinos s/n, 29071 Málaga, Spain; elenapjimenez@uma.es; 2 University School of Tourism Iriarte (ULL), University of La Laguna, Paseo Santo Tomás, s/n, 38400 Puerto de la Cruz, Santa Cruz de Tenerife, Spain; almudenabarrientos@iriarteuniversidad.es; 3 Department of Audiovisual Communication, College of Media & Communication Science, Complutense University of Madrid, Av. Complutense, 3, 28040 Madrid, Spain; davidcaldevilla@ccinf.ucm.es; 4 Department of Education, University of Extremadura, Avda. de Elvas, s/n, 06006 Badajoz, Spain; 5 College of Education, Ana G. Méndez University, Cupey Campus, San Juan, PR 00926, USA

**Keywords:** reading competency, emotional intelligence, post-adolescent, interpersonal competency, affective behavior, family literacy

## Abstract

Numerous studies show that the family plays a crucial role not only in the education of children but also in the acquisition of skills in the process of teaching and formal learning, especially in their reading competence. Furthermore, within the family, studies point to the basic role of the mother as the main axis of both educational and social teaching. The approach of this research aims to analyze whether maternal habits can influence the reading competence of their children. On the other hand, numerous studies point to the relationship between reading skills and emotional intelligence. Its inclusion in the equation of this construct can give information that will nuance the learning process in this evolutionary process. Thus, in this research, the objective is to establish the existence of a relationship between maternal reading habits with respect to reading competence and emotional intelligence in post-adolescents. Four-hundred-twenty first-year university students participated between the ages of 18 and 20 (43.8% men and 56.2% women) from the Andalusian universities of Granada, Malaga, and Jaen, all of them located in areas of medium socio-cultural context. Moderate mediation and factorial ANCOVA analyses have been carried out. The results point to the fact that the profile of the post-adolescents with the best score in reading competence also scores better in emotional intelligence and their mothers are those who score highest in reading habits. Thus, the role of the mother within the family is even more important than it appears in a society that seeks parity. New forms of work–family conciliation are necessary in order not to break the mother–child bond.

## 1. Introduction

Historically, the family has been the first social, economic, and cultural nucleus in the development of the human being. However, also, and above all, in the teaching-learning process. This is stated by UNESCO in objective 4 of the UNESCO Agenda for Sustainable Development [1]. In this learning process, reading skills play a role that is not only basic but also structural, in order to generate other knowledge and to improve school performance [2]. In Spain, for example, already in high school, it is included as one of the fundamental objectives: Royal Decree 1105/2014 of December 26th, which establishes the basic curriculum of Secondary and Compulsory Education and the High School [3], much more in a decisive and voluntary stage for the human being, the educational improvement in the university. Thus, reading skills associated with the development of reading ability have become a current subject of study through different procedures [4,5,6,7,8,9,10] since they are also a necessary basis for the process of socialization of the student body [11,12], as well as for better performance in the university [13].

Reading has become social, as can be perceived by contemplating the contingent of tweets, posts, or publications generated by the hundreds of hashtags related to books and authors [14]. In the broadest sense, we understand that these educational aspects are part, because they are included, of the values promoted by traditional education, such as friendship, companionship, help, etc. [15,16], and especially social skills [17,18] that are conveyed by reading. Reading is not an activity that is performed mechanically, but requires our full attention and involvement. It is not an easy task, so you need high motivation and dedication. As it is not an innate activity in people, it is an exercise that, with practice, effort, and the use of certain strategies, can help individuals to achieve a complete mastery of it. To achieve this, it is essential that the reader is motivated and attracted by this task.

In this sense, the family and the environment that facilitates the practice of reading is understood as a study variable in the development of the reading habit, as well as that of comprehensive reading [19,20,21,22], and can even help with higher skills [23,24], although reading competence is not directly reflected in the laws of education in Spain [25].

Within the family nucleus, it is usually the grandparents and parents who facilitate contact with reading in the first instance, and it is the family is that motivates reading [26,27], even before primary school [28]. According to Márquez [29], reading habits can originate at home, but the role of the school is key in contexts of poverty, and the community provides little help to acquire this learning.

Minimum levels of acquisition of reading and writing processes must be established in a collaborative way between families, the child’s family environment, and the school [30], which is the basis for development and subsequent access to university [31]. It should be noted that the existence of studies that affirm that human development [32] as well as cognitive and emotional abilities and capacities depend on the social rooting or uprooting of the family and the support provided by government institutions [33,34,35,36,37]. Thus, in the implementation of a family reading program, Mora et al. [38] showed that students with reading difficulties improved if their families participated in the process of acquiring this competence, as also mentioned by Miller et al. [39].

## 2. Background

In the university environment, a space where a significant percentage of post-adolescents in the Western world are located, and a focus of interest of the studied problem, results of works have been published that show that university students prefer to read at home rather than at university [40,41], regardless of whether the format is digital or traditional, another variable worth studying [6,25,42,43]. Literary reading has become a social activity, especially for adolescents who are followers of children’s and young adults’ literature (LIJ) [44]. Proof of this is the appearance of booktubers, thematic social networks dedicated to reading and writing, LIJ labels (or hashtags) and an infinite number of fanfictions [45].

With respect to the role of mothers, it has been demonstrated that they influence the cognitive development of their children [46,47,48,49,50]. Thus, it is shown that the higher the educational level of the mother, even with a “slightly higher” educational level than that of the father [19], the children improve their academic performance [51]. It is not in vain that the mother devotes more time to helping with homework [52]. It has been proven that there is a latent problem, but one that has been little studied, which reveals that mothers are an essential element in the learning development of their children [7]. Larrañaga and Yubero, in a study carried out on primary school students, indicate that the children perceived that the adults in their environment liked to read, but that it was the mother who dedicated more time to it, after the figure of the teacher, consolidating these maternal reading habits that occupy the present study. However, is this influence maintained in advanced ages, when the child has left behind adolescence to become a post-adolescent? Sánchez Cobarro [53] studies the emotionally intelligent organizations providing a theoretical framework to the emotional progress in adults.

There are also numerous studies that combine reading skills and emotional intelligence in one way or another [37,54,55,56,57,58], since emotional intelligence not only improves the relationship between students and teachers, but also their work habits [7,59,60,61] and their general academic performance [62,63,64] and their motivation for reading in particular [20,28,65,66]. Training teachers in emotional education should be considered fundamental in order to be able to act as guides who also teach about emotions and social well-being [67,68,69,70]. Studies have been carried out that show a direct relationship between some factors and others, pointing to the notable influence that emotions have on the performance and general development of students in educational centers [71,72], which leads us to consider the training of students in emotional skills as necessary [73,74,75,76].

In this sense, it is worth noting the words of those responsible for the concept of emotional intelligence, Salovey and Mayer [77], when they indicate that literature is probably the first home of the emotional intelligence. Recently, Barrientos-Báez et al. [78] defined emotional intelligence as the ability to control and positively manage one’s own emotions and those of others in any scenario where experiences and changes occur as part of the personal learning process. There is no doubt that reading is a basic tool for the development of emotional competencies [52,60,79,80,81,82,83,84,85,86] and that the role of the mother in the choice of her children’s readings favors a greater emotional attunement. Aram and Aviram [87] note that the use that mothers make of the language of the state of mind during reading facilitates the understanding of the world [88,89].

The mother’s role as the central axis of her children’s reading education places her in a privileged position within the family bosom and, by extension, in the reading and emotional learning process of her offspring. Given the importance of this situation, the main objective of this study is to analyze how mothers’ reading habits influence the reading competence of their children through emotional intelligence.

We cannot fail to mention the risks that exist in the formation and behavior of post-adolescents, whose limits are set by authors such as Casado et al. [90] or Fernández-Mojica and Barradas [91] in terms of their area of development or their personal [92] or geographical situation [93].

## 3. Materials and Methods

### 3.1. Objectives

The general aim of this study is to analyze the connection between maternal habits and their possible influence on the reading competence and emotional intelligence of her children. In this sense, we are basically pursuing two objectives: Objective 1 (PO1), to determine whether maternal habits influence the reading skills of children and whether this is reflected in the social and educational environment, Objective 2 (PO2), to establish the relationship between reading skills and emotional intelligence, and to verify whether these maternal habits also influence the emotional intelligence of her children. We consider that post-adolescence is the best time to establish these relationships as it occurs at the end of the entire evolutionary process, both social and educational, that has developed during childhood and adolescence.

### 3.2. Participants and Sample

Focusing on the post-adolescence stage, this research was planned with a quasi-experimental design of a longitudinal nature using a sample of university students in their first year of studies (they enter at the age of 18). The protocol for this field work was in line with the guidelines of the Ethics Committee of the universities to which the researchers belong. The descriptive studies conducted in Spain do not require institutional approval, but in our case, we comply with the Codes of Good Practice for Research on Human Beings. For their part, the participants gave their informed consent in accordance with the Declaration of Helsinki. All the ethical parameters used for research with questionnaires on human beings were met: Communicated by the right to information in addition to the protection of personal data, confidentiality guaranteed, policy of not discriminating for any reason, intervention free of charge, and that the study can be abandoned at any time, etc. The application of the instruments used fully guaranteed confidentiality and anonymity.

The sample was made up of 420 post-adolescents (*N* = 420), all of them university students from the Faculties of Educational Sciences (44% men and 56% women). Three universities participated: Malaga, Granada, and Jaen. The centers were located in areas of medium socio-cultural context. Since all the subjects were of similar age, educational level, and common interests (university students from the educational field), and a similar socioeconomic context (belonging to public universities with students from very similar fields), the maximum homogeneity possible was achieved in the sample for the study. The reasons for having chosen post-adolescents were their ability to respond with greater precision than children to the questions on a research instrument and because, being university students, they had developed the entire compulsory education stage, which made the study more homogeneous.

### 3.3. Instruments and Procedure

The instruments used in the field work were the following:

*Test of Reading Competence* (ComLEC) [94]. This questionnaire is designed with five texts, of which three are continuous and two are discontinuous. It presents a total of 20 questions generated according to PISA 2000 guidelines. The length of the texts ranges from 274 words to 426, and they are mainly expository and argumentative fragments, in the case of the continuous ones, and diagram and graph with a minimum of 130 words in the case of the discontinuous texts. If the questions are answered, they are closed, with the type of multiple-choice predominating. The twenty questions are divided, following PISA guidelines, into information retrieval, integration, reflection. The ComLEC reading competency questionnaire obtains an internal consistency of (α = 0.79) and corrected homogeneity indexes of items with values between 0.41 and 0.48. Despite the fact that this test has been designed for 15-year-old adolescents, the texts and questions used are perfectly valid for higher age ranges since the type of texts and questions suggested by PISA have been used for both school students and adults.

Spanish version of the *Wong and Law Emotional Intelligence Scale* (WLEIS) [95,96]. This questionnaire consists of 16 questions with a seven-point Likert answer format which evaluates EI in the organizational environment, structured in four dimensions: (1) Evaluation of one’s own emotions, (2) evaluation of the emotions of others, (3) use of emotions, and (4) regulation of emotions. It allows obtaining a total score where the higher the score, the higher the EI. The test is administered to subjects from the age of 16, so it is suitable for the present study. It shows satisfactory internal consistency values measured by Cronbach’s alpha coefficient (α), ranging from 0.83 to 0.90. In the study sample, the results are similar to those of the original version, obtaining internal consistency values for the four dimensions between 84 and 0.89, as well as a α = 0.89 for the total score. 

*Ministry of Education Reading Habits Questionnaire*. Published by the Ministry of Education, Culture, and Sport of the Spanish Government (MECD) in 2019 [97]. This test consists of 50 questions addressed to secondary school students. The test seeks to obtain information about the socioeconomic situation of the respondents, not only about their reading habits but also about the reading context in which they develop. In this specific research, items directly related to reading habits and also the socioeconomic situation of the family have been chosen, that is, questions 6 to 20 about present have been selected: Is considered a reader, the father reads, the mother reads, give books away, ask about reading (father and mother), recommend reading, there are books at home, talk about books (mother and father), recommend teachers, use of the library, attend the book fair, awareness of the taste for reading, approximate income in the family nucleus, teachers encourage reading, parents encourage reading, and the desire to read more. It resulted in questions focused on the mother figure in order to obtain precise and concrete and non-generalist results that diverted the focus of attention from the main objective that is based on reading comprehension, the mother figure, and emotional intelligence.

The procedure followed began with sending an email to university professors who are members of the Spanish Association of Reading Comprehension (Asociación Española de Comprensión Lectora) to offer their students the possibility of participating in the study. The research team informed the professors who agreed to participate of the web address where the questionnaires were located (http://testcl.comprensionlectora.es/). It contains several questionnaires from which the above-mentioned ones have been selected for this study. All of them were made in class, in a digital classroom (all the students had a computer individually), controlling the times by the teachers responsible for the subjects, during the two-hour theory classes. The data were already available during the year 2019.

## 4. Results

### 4.1. Data Analysis

Three data analyses have been carried out with the IBM SPSS tool version 23. With respect to the moderation analysis, the total score in reading competence is taken as the dependent variable, the independent variable is sex and, finally, the total score in emotional intelligence is used as the mediating variable. A moderate mediation analysis is also carried out in which the mother’s reading habit variable is introduced as a modulating variable. Finally, a 2 × 2 factorial ANCOVA has been carried out, where the dependent variable is the total score in reading competence, the factors are the mother’s reading habit (no and yes), and sex (male and female), and the covariate is the score in emotional intelligence.

### 4.2. Moderation Analysis

The moderation analysis aims to assess whether the relationship between sex and reading competence depends on maternal reading habits, as noted in the first objective. This analysis determines whether the relationship between sex and reading competence changes at different levels of maternal reading habits as a moderator. The moderator (*w*) is included in the analysis as an interaction term [98]. The results indicate that the effect of interaction is not significant (*β* = 0.81, *p* = 0.19), showing that the relationship between sex and reading competence does not change at the different levels of maternal reading habits.

### 4.3. Moderated Mediation Analysis

**Figure 1:**
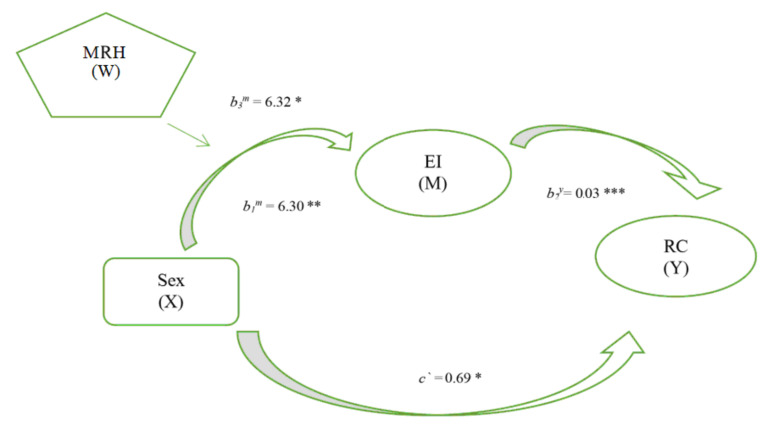
Moderated Mediation Model. Note. * *p* < 0.05; ** *p* < 0.01; *** *p* < 0.001. X = Independent variable, M = Mediating variable, W = Moderating variable, Y = Dependent variable, EI = Emotional Intelligence.

In this case, the analysis seeks to verify whether there is a relationship between reading competence and sex mediated by emotional intelligence and, in turn, whether this relationship changes according to the mother’s reading habits score, as indicated in the second objective. The results show that all the effects are statistically significant (Figure 1).

For its part, the moderate mediation index (b3^m^ × b2^y^) quantifies the indirect conditional effect of the sex variable on reading competence, which indicates how the effect of sex on reading competence through emotional intelligence changes according to the values of maternal reading habits. To test its statistical significance, the bootstrapping method was used with 5000 bootstrap samples to construct the 95% confidence intervals. The moderate mediation index was statistically significant (b3^m^ × b2^y^ = 0.29) since 0 was not found in the 95% bootstrap confidence interval (0.08–0.47). The data show that university students with higher emotional intelligence and whose mothers score higher in reading habits are those who obtain greater reading competence.

### 4.4. Comparative Analysis

Finally, and with the intention of examining the differences in reading competence according to the mother’s reading habits, sex, and emotional intelligence, an ANCOVA 2 × 2 was carried out, with the factors to be taken into account being sex (male and female) and the mother’s reading habits (yes and no), and the covariate being emotional intelligence. The data show that the covariate IE has been statistically significant *F*(1497) = 22.01, *ω*^2^ = 0.61, *p* < 0.001, which indicates that it maintains a linear relationship with CL. The main effect of the sex factor has turned out to be statistically significant *F*(1497) = 4.33; *ω*^2^ = 0.33, *p* < 0.05, indicating that girls show a higher adjusted average in CL (M adjusted = 14.58) than boys (M adjusted = 13.55). Likewise, the main effect of the HLM factor has also been significant, *F*(1497) = 4.73, *ω*^2^ = 0.33, *p* < 0.05, showing that students with higher CL scores are those whose mothers demonstrate greater reading habits.

**Figure 2:**
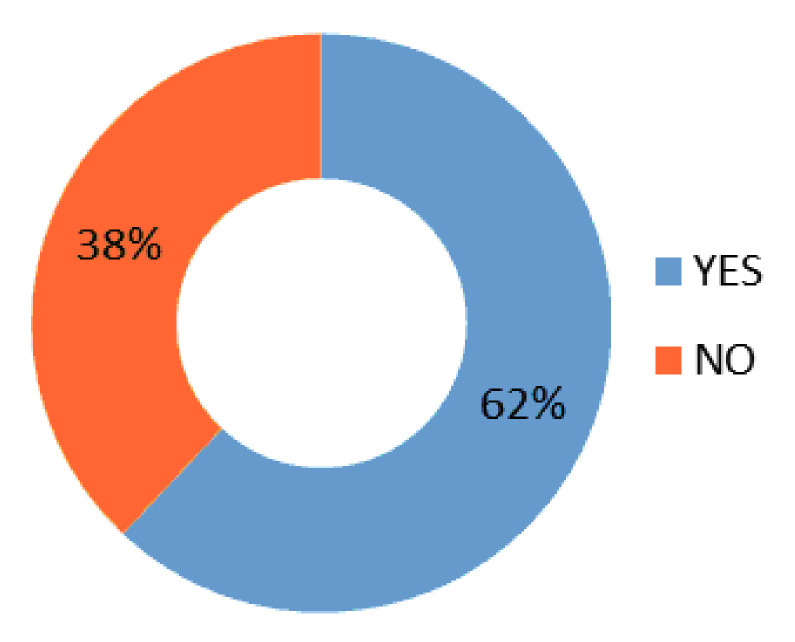
Are you currently reading a book?

These results are consistent with those obtained in previous analyses since they confirm that girls have higher CL than boys regardless of the mother’s reading habit. Other questions asked were: Are you currently reading a book? (Figure 2). Sixty-two percent of the respondents answered affirmatively when asked if they were reading any work. In contrast, 38% said they were not immersed in reading any book when they took this questionnaire.

**Figure 3:**
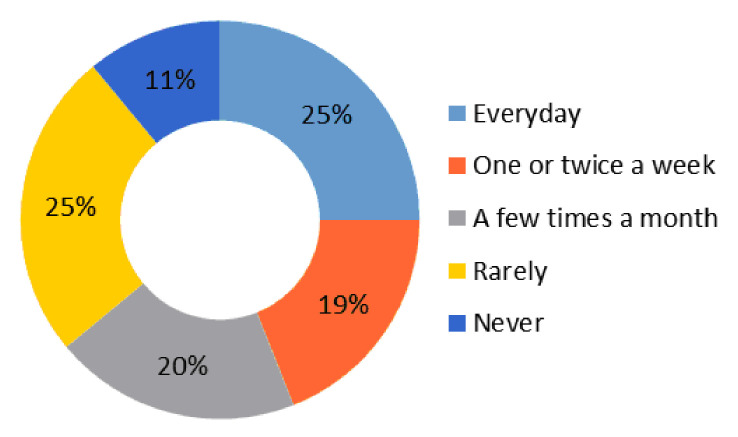
How often do you read?

Twenty-five percent of the university students interviewed claim to read daily, followed by 19% who admit to doing it once or twice a week and 20% who do it a few times a month. In short, these data reflect that 64% of the surveyed students include reading among their frequent activities. This result is acceptable, and generally positive, since it indicates that more than half of the respondents value reading. On the contrary, a worrying 36% say they never read (11%) or almost never (25%) (Figure 3).

**Figure 4:**
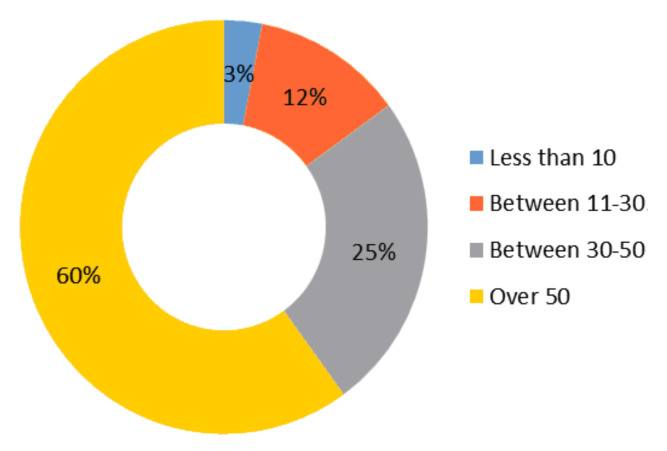
About how many books can you find at home?

Regarding the data provided by the students surveyed on the number of books they can find at home, it is striking that the vast majority, specifically 60% of those investigated reveal that the family library consists of more than 50 books, followed by 25% who say that the number of books present in the home ranges between 30 and 50 books. The most worrying data affects 12% of those surveyed who place between 11 and 30 the works these students can find at home. Parents, the family, are clearly a great example for their children, and they can determine whether their children are interested, or not, in reading (Figure 4).

## 5. Discussion

University students who claim to be readers as a way of life (who do not read out of obligation, have non-text books, or have an established reading habit) obtain better grades in general in reading competence, according to a study by Jiménez [99].

Thus, for higher levels of emotional intelligence, better data are also obtained in reading competence (IE = 5.62 with CL = 17.65) and, on the contrary, at lower levels of reading competence, a worse score in emotional intelligence (IE = 4, 44 with CL = 12.89). In the study, they highlight that it is the youngest university students (18 years old) who obtain the best reading skills. This may be due to the competitiveness that high school students have to assume in the face of the entrance exam that forces them to read and concentrate on studying. In addition, where reading habits are related to emotional intelligence, it is confirmed that they also maintain a close relationship since students with higher emotional intelligence are those who show a more ingrained reading habit (HL = 2 with EI = 5.62 ) and vice versa (HL = 0 with IE = 4.44). In IE, best scores revolve around one’s own perception of emotions, not so much those of others. Thus, respondents think or feel that they are happy, in general, that they are good observers and that they have a great capacity to encourage themselves. It is shown that there is a direct relationship between university students who better understand their own feelings and feel happier and a reading competence with the highest scores. This is probably due to the fact that culture influences the emotional adjustments of individuals and their emotional perceptions [100], and a good interpersonal and psychological functioning of individuals [101], although the fact that readers are happier is already pointed out by a study by the University of Rome [102].

It is found that not only is reading competence directly related to EI, but reading habits play a fundamental role in this relationship. Moreover, given that the observed reading is playful, merely literary, the data suggest that reading literature for pleasure can be the natural way to train reading competence, without the need to carry out specific activities with literary texts.

It is worth considering, for future research, which specific aspects of emotional intelligence influence reading competence or the tendency to create a habit from reading, as well as whether by training reading competence, better scores in emotional intelligence can be obtained and vice versa [55]. This could imply an improvement in academic performance if the educational centers further strengthen the role of psychologists as counselors on collective routines for the improvement of emotional intelligence.

There are many studies that relate the reading habits of parents and their influence on their children. The main research was developed in the 1970s, when the relationship was already established [103,104,105,106]. Over the past few years, the results have been confirmed by many other important contributions [107,108,109,110,111,112,113].

There have been fewer contributions, however, that have highlighted the mother figure in the influence of reading habits. The main studies that have obtained results in part similar to ours are those of Bus and Van Ijzendoorn [114], Bus et al. [115], and Weigel et al. [116]. However, these are works that did not contemplate, for example, the impact that ICT can have on reading habits.

It should be taken into account, as several authors have shown [117,118,119], that the impact of the current digital paradigm is one of the factors that also significantly influence reading habits, comprehension, and competence. Nor can it be forgotten that ICTs are not exclusively technologies and multimedia media, also contain large volumes of text that imply the need to read, for example, most Internet websites or social networks [117]. It would be very interesting to undertake a study that takes into account this new reality in which parents often do not perform as well as they do in traditional print media.

In addition, there are many other factors that influence reading habits. There are many contributions, such as those of Ogunrombi and Adio [120], McKool [121], Hughes-Hassell and Rodge [122], Knoester [123], or Celik [124], who establish a detailed list of these. In this work, among all of them, all that implies emotional intelligence and at the same time collaboration with parents in this objective is considered fundamental, especially the influence and reading habits of the mother and sharing them with the family [111,112,113,114,115,116,117,118,119,120,121,122,123,124,125].

Especially in the university stage and post-adolescence, the relationship between all these factors and the quality of education represent a backbone of academic success materialized in student learning outcomes. Many of the current educational policies, such as those of the European Union, influence these aspects. It can be said that in addition to parents, teachers also act as guides in learning emotions and other personal skills that, in some research on emotional intelligence and education, are clearly identified. There is a need to manage intervention programs that allow the development of emotional intelligence skills both at home and in universities [126,127]. It cannot be ignored that the processes of educational change are very slow but inevitable, and in the case of emotional education, they are indispensable [78].

All these variables are cause for concern both at the national and supranational levels. The European PISA program, for example, analyzes the results in terms of effectiveness obtained by the educational systems implemented by each country during a determined period of time. The results had an impact on and influenced the policy and ministries responsible for education in many countries [128,129].

It is not surprising, therefore, that the reading ability of students is one of the main focuses of attention for the European Higher Education Area because a large number of academic results derive from correct reading comprehension [130,131,132]. We are talking, in general, about one of the main educational objectives worldwide.

Regarding the limitations of this study, it should be noted that it would be interesting to expand the sample in future research, ideally to the entire national educational system, in order to understand more precisely how constructs evolve in human development towards reading from the beginning of primary education to the end of university studies, which could help to understand the current pros and cons that facilitate or improve the cultural level of people. Finally, from this starting point, and in the specific case of Spain, where we have focused, it would be interesting to consider in future research the possible relationship between consumption of the literature through the reports of the Publishers Guild of Spain, for example, and its relationship with socioeconomic factors, such as the human development index (HDI) beyond per capita income (PCI) or gross domestic product (GDP) as factors that measure social welfare.

Another limitation of the study is that it was not developed from a longitudinal design, instead of the non-longitudinal descriptive one finally carried out, which would have been very interesting for the complementary data it could have provided. However, there are other recent studies [133,134] that have done so using the same MECD instrument [97] that we used, so we decided to focus it more specifically on the selected object of study.

## 6. Conclusions

In this research, we found data that indicate that there is an influence of mothers through their reading habits on the development of their children’s reading competence, that is, they not only transmit the passion for reading, but also influence the results of the reading competition. However, women show better results than men in reading competence, regardless of maternal influence, which is why the natural inclination of the female sex towards the world of reading stands out since reading is the natural way to train the reading competence. However, it is worth noting the primary role of mothers within the family, given the positive influence shown by both the emotional level and the fact that their children are readers, as other studies in other age ranges have already pointed out. Perhaps new legal guidelines, less superficial than the current ones, on the reconciliation of work and family, as in other European countries, would be an interesting proposal for the improvement of education from childhood.

In addition, reading competence and emotional intelligence fluctuate, showing a linear relationship, which shows the influence of one on the other, since the results show that female university students give a better average than male university students in reading competence, controlling for the emotional intelligence variable. On the other hand, the reading habits of the mothers show a directly proportional influence on the reading competence of the children of both sexes, that is, the greater the reading habit of the mothers, the greater the reading competence of the children.

From this study, the conclusion is drawn that EI intervenes in the optimal development of the personality and constitutes a fundamental axis for the sustenance of aptitudes, attitudes and, finally, of the individual’s relationship with others. At the international level, this research corroborates the study conducted by Rabia [135], which shows that emotional intelligence is important for the professional adaptation of university students and post-adolescents in general.

Women show better reading competence as they acquire greater emotional intelligence, but this occurs independently of their mother’s reading habits, i.e., college students score significantly better than children in general. However, according to gender, the reading habits of mothers indicate a significant effect on reading competence by controlling emotional intelligence, thus, females score better on reading competence than males.

It is not intended to retrospectively analyze a child’s memory of his mother’s reading habits. This research measures the reading habits of mothers at the time of the survey.

It is intended to analyze reality at the present time, and therefore, the universe of the study is made up of university students, students whose achievement has been to continue their studies at a university. Therefore, it is a random sample in a specific and unbiased universe.

The limitation of the study that would be interesting to expand in future research refers to the breadth of the selection of the sample from the entire educational system, in order to understand more precisely how the constructs evolve in the development of the human being from the beginning in elementary school until they finish their studies at university, which could help to understand the pros and cons of the current educational system.

Finally, from this starting point, it would be interesting to raise in future research the possible relationship between the consumption of literature through the reports of the Publishers Guild of a country, and its relationship with socioeconomic factors such as the Human Development Index (HDI) beyond the per capita income (RPC) or the Gross Domestic Product (GDP) as measurement factors of social welfare.
